# Beneficial Treatment Outcomes of Severe COVID-19 Patients Treated Entirely in Primary Care Settings With Dexamethasone Including Regimen—Case Series Report

**DOI:** 10.3389/fphar.2021.684537

**Published:** 2021-08-12

**Authors:** Damir Vukoja, Andrea Jurić, Zdravka Erkapić, Tomislav Pejić, Željko Zovko, Josipa Juričić, Jelena Pejić, Matea Ćorluka

**Affiliations:** ^1^ Grude Health Center, Grude, Bosnia and Herzegovina; ^2^ Institute for Public Health of West Herzegovina County, Grude, Bosnia and Herzegovina; ^3^ Ministry of Health, Labor and Social Welfare of West Herzegovina County, Grude, Bosnia and Herzegovina; ^4^ Department of Emergency Medicine, University Clinical Hospital Mostar, Mostar, Bosnia and Herzegovina; ^5^ Department of Dermatology and Venerology, University Clinical Hospital Mostar, Mostar, Bosnia and Herzegovina

**Keywords:** COVID-19, coronavirus, SARS-CoV-2, dexamethasone, corticosteroids, primary health care, treatment

## Abstract

Even in 2021, coronavirus disease 2019 (COVID-19) remains a major global health concern, especially in developing countries. The burden of this disease, caused by severe acute respiratory syndrome coronavirus 2 (SARS-CoV-2), which affects not only primary respiratory but also other organ systems, keeps rising as the pandemic continues. Primary health care centers are the first line where COVID-19 patients are managed and should be able to manage the vast majority of them successfully. In this paper, we present a case series and concept of beneficial management of even deteriorating and severe patients treated entirely in our primary health center. The management is based on well-timed and rational dexamethasone use, as well as on various other pharmacological and nonpharmacological treatments and interventions, and is supported by provided statistical data. According to the presented experience and positive outcomes achieved, it seems that even deteriorating and severe COVID-19 patients can be treated successfully to some extent or even completely in primary care settings. This kind of approach could be particularly beneficial in conditions of overload of higher-level health care institutions.

## Introduction

With the end of the year 2020 (as of December 31), more than 81 million cases of coronavirus disease 2019 (COVID-19) were confirmed worldwide with a death toll reaching almost 1.8 million and a global case fatality rate (CFR) of around 2.2% (World Health Organization, 2020b). In a little over a year since it first appeared, this disease caused by severe acute respiratory syndrome coronavirus 2 (SARS-CoV-2) emerged as the leading global threat of the moment by affecting almost every country and changing everyday life routine. Health system overload, economic decline, and an increase in the rate and severity of mental illness are just a few indicators of the burden of this pandemic (Dubey et al., 2020; Goulabchand et al., 2020; London et al., 2020; Yelin et al., 2020; Yusuf and Tisler, 2020).

According to emerging literature, the clinical presentation of COVID-19 can range from asymptomatic infection to mild respiratory illness, all up to more severe conditions affecting one or more organ systems requiring hospitalization and even intensive care unit admission. However, a major concern and treatment challenge correspond to often extensive viral pneumonia and severe respiratory illness accompanied with it. Regardless, there are some disparities in relevant literature in terms of criteria for classifying the illness as “severe.” Namely, while the US National Institutes of Health (NIH) considers COVID-19 patients with a saturation of oxygen (SpO_2_) ≤93% along/or with the fulfillment of other criteria like dyspnea or respiratory frequency ≥30 breaths/min as “severe,” the WHO’s living guideline sets the limit of saturation at <90% to classify patient as severe. But, current data agree that most patients are asymptomatic or have mild disease, with a tendency of older adults, men, obese, and patients with other comorbidities to be more susceptible to severe illness (World Health Organization, 2020a; Cuschieri and Grech, 2020; Patil et al., 2020; Wiersinga et al., 2020; Dehingia and Raj, 2021; National Institutes of Health (NIH), 2021).

Despite many treatment options with proven or promising benefits that appeared so far (including antivirals, corticosteroids, other anti-inflammatory agents, immunomodulatory agents, antibodies, and others) and available living treatment guidelines, the therapeutic management of patients with COVID-19 still remains the area of uncertainty (World Health Organization, 2020b; Sahebnasagh et al., 2020; Wiersinga et al., 2020; Asselah et al., 2021; National Institutes of Health (NIH), 2021). It is a fact that mortality related to COVID-19 drops as time passes, but it is still unclear how much of that reduction can be attributed to better managing of these patients (Fan et al., 2020; Preskorn, 2020). And yet, the mortality rates are still high considering the prevalence of the disease and the overall number of deaths as a consequence (Ghayda et al., 2020; Sorci et al., 2020). Considering all the above mentioned, it can be said that treating and managing these patients still poses a challenge to health professionals, especially in low to middle resource countries/regions (Sorci et al., 2020).

Indeed, the country’s income status usually impacts to a significant extent the national health care system quality, especially in these modern times more than ever. This means that the start positions and possibilities to respond and adjust to new conditions such as this pandemic vary among different countries. In the case of COVID-19, this also implies the concern with disparities in vaccine availability among countries and regions to a point where it becomes the question of ethics and human rights (Beyrer, 2021; Lambert, 2021). Accordingly, as Bosnia and Herzegovina belongs to middle-income countries and is among the weakest European economies, it is clear that its health care system found itself in an unenviable position during the COVID-19 outbreak. This is especially true considering the postwar consequences that may still be taking their toll in the present to a significant point (Sabes-Figuera et al., 2012). Therefore, heads of institutions of all three levels of health care had to put a lot of knowledge and effort into adjusting the health care system’s functioning in the best possible way given the low resources at their disposal. And, most importantly, these conditions forced not just corresponding executives but also “first line” physicians to improvise in many ways (Turner et al., 2019; Chowdhury et al., 2020; Kovacevic et al., 2021).

As a result, despite all the efforts and resourcefulness, by the end of the year 2020, there were 110,985 confirmed cases of COVID-19 in Bosnia and Herzegovina with a CFR of almost 3.7%, respectively (World Health Organization, 2020b). Based on these data, Bosnia and Herzegovina counts among the European countries with the highest mortality rates due to COVID-19 (University of Oxford, 2020). Nevertheless, there were, and still are, significant differences in CFR values among different administrative units within the country (Institute for Public Health of Federation of Bosnia and Herzegovina, 2020). Divergency in mortality rates seen among different areas in the country may be attributed to many factors such as demographic, economic, political, etc. However, this phenomenon probably depends largely on diverse local/regional health care system organization and approach of primary and higher health care institutions to its patients, as well as to different therapeutic approaches and management toward these patients by medical professionals. For instance, the municipality of Grude as part of West Herzegovina County stands out among others with a very low CFR of <1% despite the high prevalence of the disease (Institute for Public Health of Federation of Bosnia and Herzegovina, 2020). Hence, as conscientious medical professionals from the Health Center Grude (which is the primary health care institution in charge of Grude municipality patients), we were obliged to share our experience, work scheme, therapeutic approach, and management of COVID-19 patients, which seem to be overall beneficial.

## Cases

Among many other cases of successful management of severe COVID-19 patients at this primary health institution, the most suggestive and representative ones were selected and presented in the following. The patient was considered as severe if the criteria by NIH definition were met (National Institutes of Health (NIH), 2021). Similar to those here presented, the other severe and some “almost severe” patients were managed in like manner too by following and applying current and updated treatment guidelines as much as possible (World Health Organization, 2020a; The National Institute for Health and Care Excellence (NICE), XX; Chow et al., 2020; Gautret et al., 2020; Wiersinga et al., 2020; Bottari et al., 2021; Gyselinck et al., 2021; National Institutes of Health (NIH), 2021), while respecting an individualized approach.

### Case 1

**FIGURE 1:**
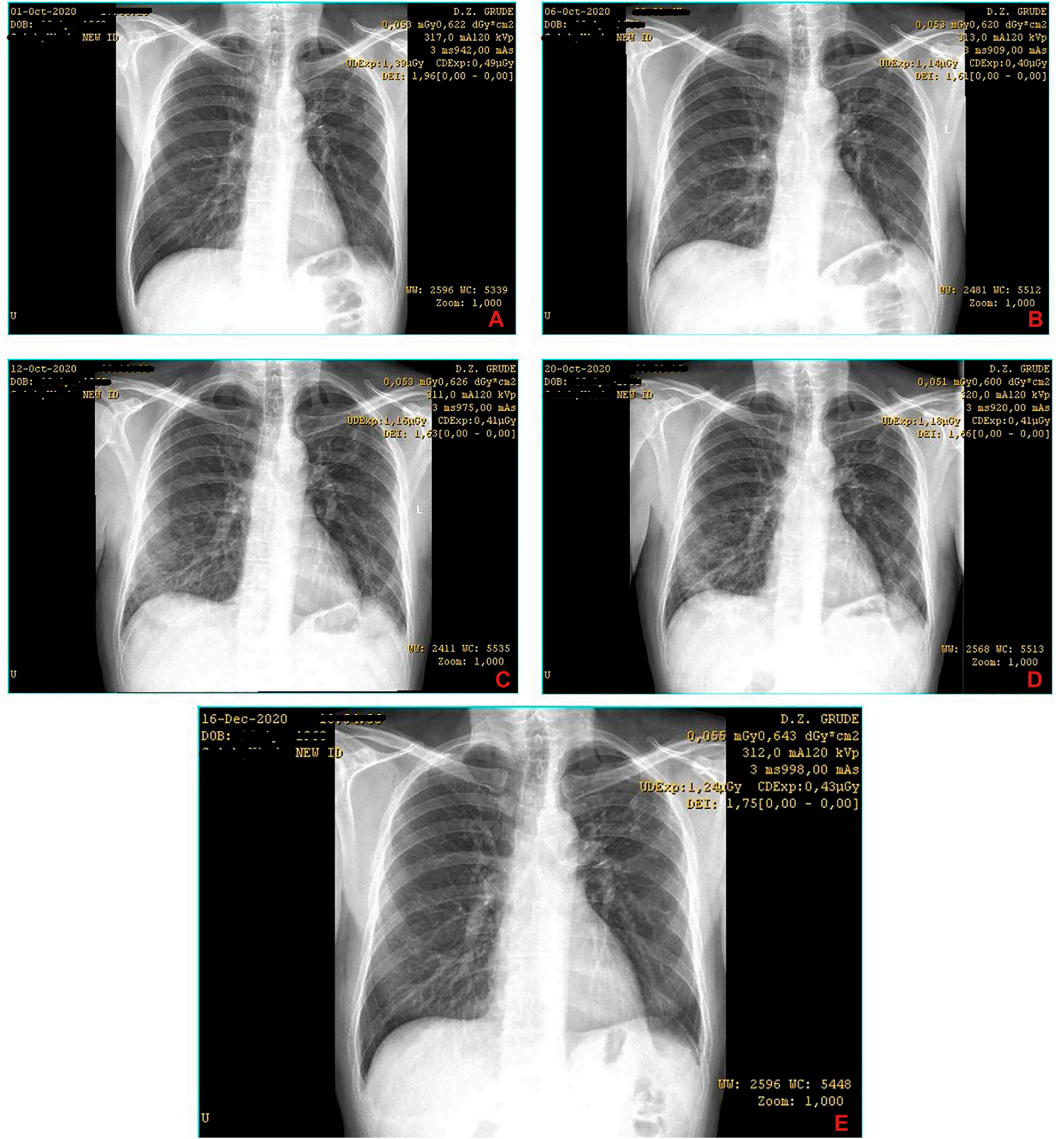
Series of chest X-ray images of COVID-19 patient from case 1. The images are chronologically* arranged in a–z order (the letter mark is indicated in the **lower right** corner of each image)^§^. *The dates of records are visible in the **top right** corner of each image. ^§^Identifiable patient’s data are covered.

A 57-year-old male patient, nonsmoker for more than 10 years, with comorbidities of bronchial asthma and Parkinson’s disease, presented to a general practitioner two days after the onset of symptoms. The patient was subfebrile up to the presentation, had a dry cough, and denied any other symptoms, either new or worsened health difficulties. In the epidemiological anamnesis, there was no information of significant importance. Physical examination revealed hypokinesia and graceful body composition by inspection. Lung auscultation showed slightly decreased breathing sounds, especially in the lower fields. Blood oxygen saturation (SpO_2_) was at 98%. Basic blood laboratory analysis and chest radiograph were performed (Figure 1A), none of which showed significant pathology (except for a slight increase in granulocytes regarding lab work). The patient was advised to take symptomatic measures and was referred for a PCR test for SARS-CoV-2 the next day. The test result was positive; the diagnosis of COVID-19 was made. Having in mind the patient’s comorbidities as well as age, sex, lung auscultation finding, and other factors, treatment with azithromycin 500 mg once daily and aspirin 400 mg daily divided into two doses was initiated. An over-the-counter (OTC) supplement containing beta-glucan, vitamins, minerals, enzymes, peptides, and amino acids formulated to boost immunity and recovery was recommended. At the control appointment two days later (the sixth day from the start of the disease), there were no significant changes in symptoms or body temperature rises, the SpO_2_ was at 97%, but a control X-ray image of the chest was done to screen for viral pneumonia. Bilateral pneumonia of lower lung fields was described by a radiologist (Figure 1B). At follow-up encounter two days later (the eighth day of the disease), the patient is still subfebrile but with body temperature values lower than earlier and is still coughing but mostly when changes milieus with different air conditions (cold/warm and *vice versa*). By inspection, mild dyspnea is noticed and SpO_2_ is at 95%. At this point, parenteral therapy with dexamethasone 8 mg and crystalloid solutions 250–500 ml daily (mostly Ringer’s and saline solution) was started and received at adequate spaces for such patients at the health center. The aspirin dose was reduced to 100 mg daily. The next day, the lab follow-up was done and it showed mildly elevated c-reactive peptide (CRP) levels at 15.7 (mg/L), while liver, renal, and cardiac parameters were regular. Since it was the last day of taking azithromycin, moxifloxacin 400 mg daily was prescribed for the following period alongside probiotic containing yeast *Saccharomyces boulardii*. On the 12th day from the onset of symptoms, the patient was still dyspnoic, SpO_2_ was at 92%, his appearance had worsened, and his family member reported that he looks weaker. Control chest radiogram showed progressive dynamic of bilateral pneumonia and discrete pleural effusion in the projection of the left lateral phrenicocostal sinus (Figure 1C), while CRP value was reduced to 6.5. Hospital treatment was suggested, but the patient and his family preferred to continue the treatment at the health center under the supervision of the same doctor. Thus, the same treatment was continued but with dose deescalation of dexamethasone to 6 mg daily for following days, and administration of supplemental oxygen was started (up to 5 L/min via nasal cannula for about 90 mins a day). In the following days, the saturation slowly started to rise up; the patient was less dyspnoic and was feeling stronger. At follow-up encounter with the doctor three days later (15th day of the disease), the SpO_2_ rises up to 95%, there is no longer noticeable dyspnea, the cough was reduced, and the patient was afebrile for a couple of past days. Control lab work was without significant deviations (except the elevated total leukocyte count, which was attributed to dexamethasone use). It was suggested to continue taking moxifloxacin and dexamethasone 4 mg orally for the next four days as well as probiotics, earlier mentioned supplements, and aspirin. 20 mg pantoprazole was added to therapy, while parenteral administration of fluids as well as oxygen therapy was discontinued. At the final control appointment three days later (18th day from the onset of symptoms), the patient was feeling well; control lab work showed CRP value reduction to 0.8, while other blood parameters were normal as well, except mildly increased ALT and still elevated leukocyte count. Further, a control chest X-ray showed mild regressive dynamic of infiltrative inflammatory changes of lung parenchyma (Figure 1D). The patient was advised to continue taking probiotics, pantoprazole, and aspirin for two more weeks and was recommended to visit a pulmonologist and internist in about one month with a control chest radiograph and blood lab analysis. Approximately a month and a half later, the patient appeared at an appointment at specialists, and the control chest X-ray image was described as a normal finding by a radiologist, meaning that no scaring changes in terms of postinflammatory fibrosis were observed (Figure 1E).

### Case 2

**FIGURE 2:**
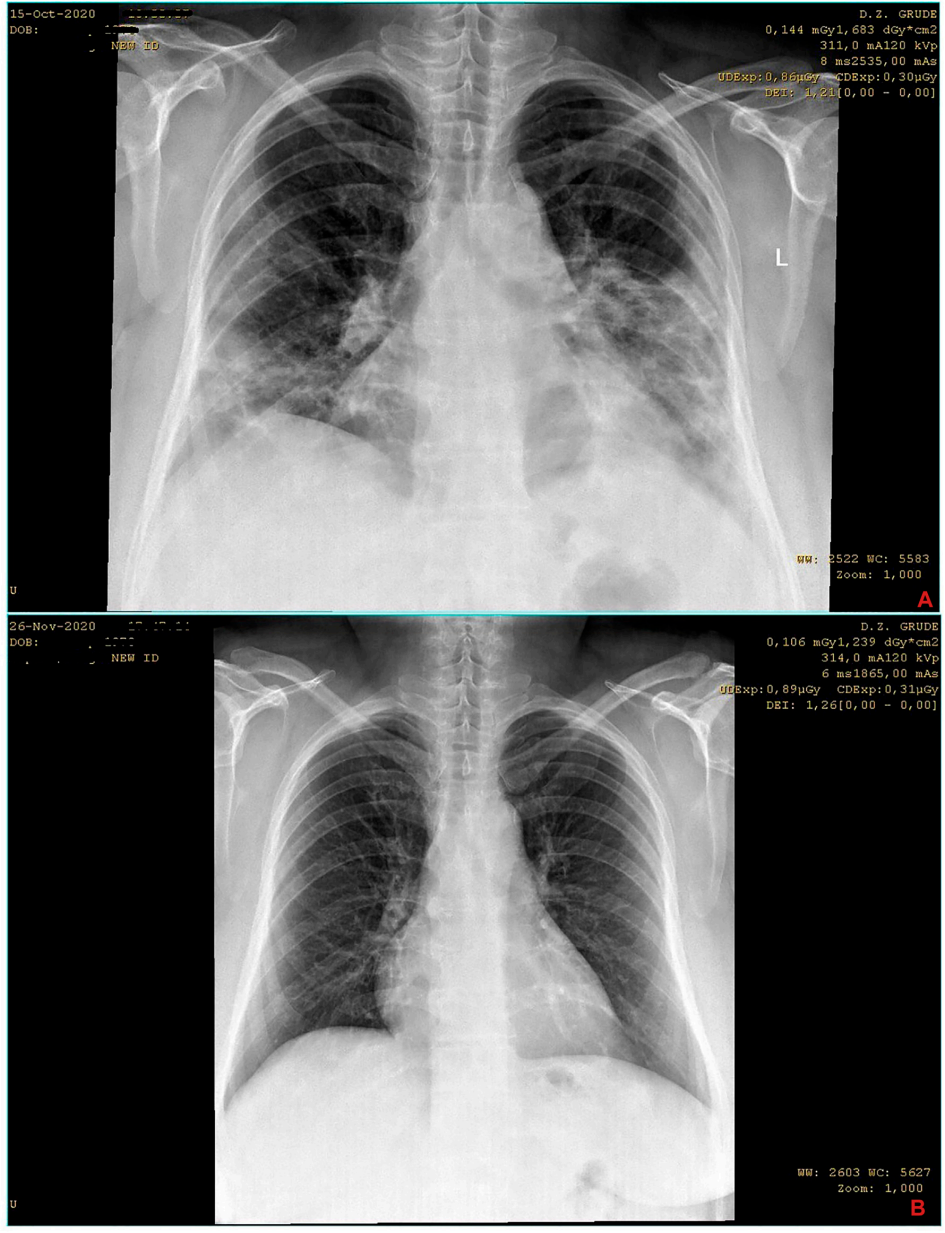
Series of chest X-ray images of COVID-19 patient from case 2. The images are chronologically* arranged in a–z order (the letter mark is indicated in the **lower right** corner of each image)^§^. *The dates of records are visible in the **top right** corner of each image. ^§^Identifiable patient’s data are covered.

A 50-year-old female patient with an extensive medical history presented to the physician’s office for being febrile up to 39°C for more than 10 days. She is a nonsmoker and her current medical problems include hypertension, hypothyroidism as a consequence of autoimmune thyroiditis, and irritable bowel syndrome, for all of which she is taking medications (bisoprolol 2,5 mg, levothyroxine 50 mg, and mebeverine 300 mg XR daily). Looking into her medical history reveals a couple of episodes of sideropenic anemia due to dysmenorrhea in recent years, gout arthropathy, an episode of pericarditis four years ago, and an episode of transient ischemic attack (TIA) 11 years ago; the patient also had a tonsillectomy in her youth due to frequent strep throat episodes and cholecystectomy approximately 10 years ago. Besides, the patient is obese and has chronic lower back issues. At the presentation, she admitted that she has been taking azithromycin 500 mg daily in the past three days “on her own.” In the physical examination, a lower blood pressure of 90/60 (mm/Hg) was detected; lung auscultation showed slightly muffled breathing sounds diffusely and small crackles in the projection of lower lung fields, while saturation (SpO_2_) was at 89–90%. Despite these decreased saturation values, the patient was not dyspnoic, and she had not had noticeable respiratory difficulties. Basic laboratory blood analysis and chest X-ray processing were done. Remarkably elevated CRP value (90.4 mg/L) and granulocytosis were detected as significant among other biochemical and blood count parameters. Equally important, an X-ray image of the chest was described as “extensive infiltrative changes of the lung parenchyma bilaterally” by a radiologist (Figure 2A). Based on these findings, it was suspected that the patient is having COVID-19. Taking into account all the above-mentioned parameters, the patient was considered to be at high risk of developing a severe form of the disease. Since she first presented to the doctor at an advanced stage, hospitalization was strongly suggested after a detailed explanation of her health condition and prognosis. Nevertheless, the patient and her family member refused hospitalization, so the treatment was started in the primary health care institution (i.e., local health center). The treatment began with parenteral fluids (crystalloids) and dexamethasone in doses of 8 mg once daily alongside oxygen therapy in the duration of a minimum of 1 h a day. It was suggested to continue azithromycin 500 mg for two more days and to add moxifloxacin 400 mg, as well as aspirin 400 mg daily, *per os*. Also, it was recommended to take the supplement mentioned in Case 1 and probiotic containing yeast *Saccharomyces boulardii*. The following day, patient had a control appointment, and a PCR test for COVID-19 was done. The patient was not reporting the deterioration of her health condition and denied difficulties with breathing. However, the blood oxygen saturation (SpO_2_) measured with a pulse oximeter was not higher than 81% despite various measurements. Although the hospitalization was strongly advised, the patient refused once again. So, the same treatment was continued for the next days; the patient continued oral therapy and came twice a day for the oxygen therapy and parenteral administration of fluids while being supervised by her doctor. In the meanwhile, the test result for novel coronavirus came positive, so the diagnosis of COVID-19 was made. During the following days, the patient was stable by saturation and general condition. At the control appointment with the doctor three days later (the 15th day from the onset of symptoms and the fifth day of the parenteral treatment initiation), the patient felt better and was afebrile for the past four days, while the SpO_2_ was up to 95%. Control lab work was done and it showed the CRP reduction to 6.4 (mg/L), while the platelets were mildly elevated (as well as leukocyte and granulocyte count, but it was attributed to dexamethasone action). It was suggested to continue with the same *per os* therapy, while the parenteral and oxygen therapy were discontinued. Dexamethasone was prescribed in the form of tablets with dose deescalation to 6 mg daily, alongside 20 mg of pantoprazole. At the follow-up appointment three days later (the 18th day from the disease onset and the eighth day of initiation of dexamethasone), the patient was feeling well and “practically healthy,” the saturation was at 96%, and she was referred to control chest X-ray imaging. The image showed “almost complete regression of the infiltrative changes along with suspected fibrose changes here and there in the lung parenchyma,” described by the words of the radiologist (this image was lost due to technical malfunction and thus not shown here). The same treatment was continued but with a lower dose of dexamethasone (4 mg) for three more days and deescalation of aspirin to 100 mg daily, which was recommended for three more weeks. It was recommended to seek pulmonologist and internist control in a month to month and a half. Later on, as part of the specialist’s examination, new control chest X-ray imaging was performed and it was described as a “normal finding” by the radiologist, meaning that no previously described fibrose changes were now visible (Figure 2B).

### Case 3

**FIGURE 3:**
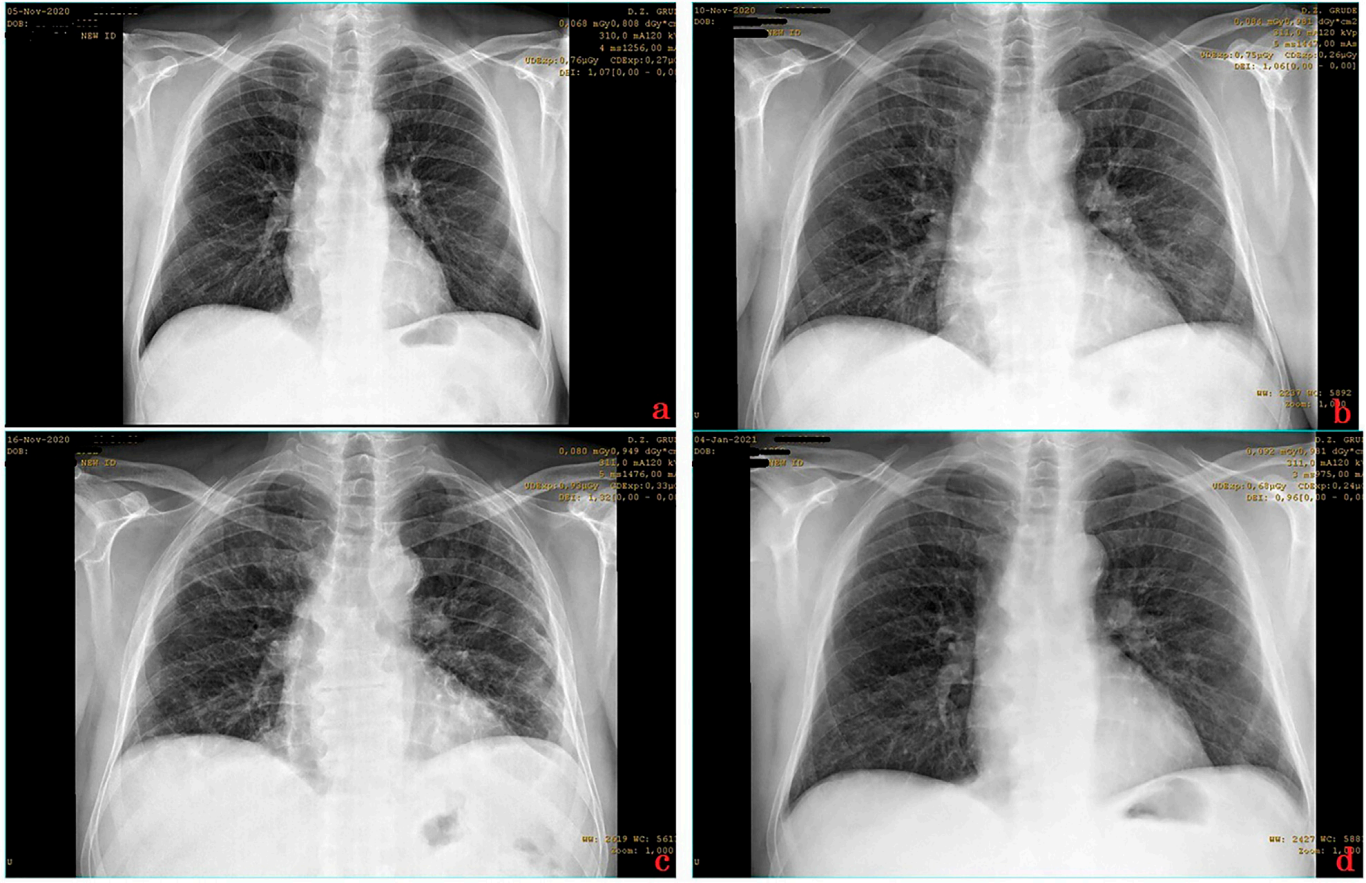
Series of chest X-ray images of COVID-19 patient from case 3. The images are chronologically* arranged in a–z order (the letter mark is indicated in the **lower right** corner of each image)^§^. *The dates of records are visible in the **top right** corner of each image. ^§^Identifiable patient’s data are covered.

A 68-year-old male patient, nonsmoker and overweight, presented to the physicians’ office for being subfebrile and having dry cough for the past four days. His medical records show a previous history of myocardial infarction and ongoing hypertension, for which he has been taking carvedilol 12.5 mg, lisinopril/hydrochlorothiazide 10/12.5 mg, aspirin 100 mg, and atorvastatin 20 mg once daily perorally for more than nine years. Patient anamnesis regarding his current condition did not reveal much additional significant data; he did not have any other developed symptoms, and his epidemiological anamnesis was nonsignificant. The physical examination revealed slightly decreased breathing sounds in the projection of lower lung fields bilaterally, while the SpO_2_ was 96%. There were no other significant findings, so the basic lab work and chest X-ray were ordered. The laboratory analysis did not show any remarkably disturbed parameter, while the chest radiograph revealed a mildly more pronounced interstitial pattern basally and toward the periphery of lower lung fields (Figure 3A). For the radiologist, this finding was most suggestive for incipient atypical pneumonia. At this point, the COVID-19 was suspected and the patient was referred for PCR testing, which later came out as positive. The treatment with azithromycin 500 mg, some herbal cough relief tablets, and an immune supplement (containing beta-glucans, vitamin D, vitamin C, and group B vitamins, as well as elements such as zinc, selenium, copper, and manganese) was initiated. An additional recommendation was to increase the daily dose of aspirin to 300 mg for a couple of following days. Two days later, on the seventh day from the onset of symptoms, the patient reported that he feels weaker and gets tired easily, his taste sense changed, and he lost appetite. At the presentation the following day (the eighth day from the onset of the disease), the SpO_2_ was notably decreased to 91–2%, and (by inspection) the patient did look weaker indeed and would even get tired while speaking. So, the control lab work and an X-ray chest imaging were done. The chest radiograph showed progressive dynamic of inflammatory changes (Figure 3B), while the blood count revealed slightly reduced total leucocytes and platelets and mildly elevated granulocytes. Among biochemical parameters, CRP was surprisingly low (5.7 mg/L), while transaminases and urea were mildly elevated. Other liver, renal, and cardiac markers were within physiological limits. Parenteral intravenous solutions were given (750 ml in total) as well as dexamethasone 8 mg and pantoprazole 20 mg through the same route. This resulted in increased SpO_2_ at the end of the therapy (up to 94%), and the patient was feeling better as well. Moxifloxacin 400 mg once daily was added to peroral therapy. The next day (day nine), the patient again looked weak, a little unstable on his feet, and was pale; discrete dyspnea was noticed and SpO_2_ was 91%. Same parenteral therapy was given and oxygen therapy was initiated (1.5 L/min through the nasal cannula for an hour and a half). Due to chronic heart disease, an internist was consulted and trimetazidine 35 mg MR twice daily was added to therapy (off-label) to support the heart function in the conditions of lower blood oxygen levels and inflammation. On day 10, the patient had similar appearance, the SpO_2_ was 90%, and dyspnea was more noticeable. The hospitalization was proposed, but the patient was not motivated at the moment. So, the treatment was continued with 500 ml of parenteral intravenous fluids, as well as 6 mg of dexamethasone, 20 mg of pantoprazole, and oxygen therapy (2 L/min). On day 11, the patient is afebrile for two days, the cough is only present when changing rooms with different air temperature or humidity, but the physical appearance is worse in terms of exhausting look and noticeable depressed mood. Hospitalization was now strongly suggested, but the patient refuses it. The same treatment was continued with the rise in oxygen flow to 3 L/min. Anxiolytic drug alprazolam 0.5 mg XR once daily was added to therapy and the importance of breathing exercises was explained to the patient. For the next two days (12th and 13th days), the patient’s condition was still poor with an even lower SpO_2_ value (88–89%), but the same treatment scheme was continued with raised oxygen flow up to 5 L/min. On the 14th day from the disease’s onset, the patient’s overall appearance and condition were a little better, and the SpO_2_ started to rise to 90–1%. Crepitations were discovered in lower lung fields by auscultation and noticeably shortened inspiration as well. Therefore, administration of intravenous fluids was reduced, and at the end of it, furosemide 20 mg was given via intramuscular route. Accordingly, oxygen flow was reduced to 3 L/min as well. Control lab work was done, and it did not reveal any new change of importance. On day 15, the SpO_2_ was 92%, the patient was feeling even better, but he was still with a reduced ability to tolerate everyday activities such as walking upstairs. Control chest imaging was performed and it showed initial regressive dynamic of inflammatory changes (Figure 3C). Since the patient’s condition improved, it was suggested to discontinue alprazolam and to reduce aspirin’s dose back to 100 mg. At the control appointment the next (16th) day, the SpO_2_ was 93%, so it was the last day of the same parenteral and oxygen therapy as the day earlier. Dexamethasone was continued perorally 4 mg daily for the next four days, as well as pantoprazole 20 mg. The patient continued to practice breathing exercises, and his ability to tolerate the exertion improved continuously with every new day. Interestingly, according to the PCR retest performed on the 18th day from the onset of the symptoms, the patient was still positive for SARS-CoV-2, which confirms that this patient’s viral load was probably very high. Approximately a week later, the SpO_2_ value was 95–6% and he was feeling almost completely recovered. It was recommended to him to seek an internist and pulmonologist control checkup in a month to month and a half. In that period, the patient was experiencing some “post-COVID syndrome” symptoms, but they were tolerable and easily manageable. Prior to specialist controls, the new chest radiograph was performed and it was described as “almost complete regression of the inflammatory changes of the lung parenchyma” by the radiologist (Figure 3D).

### Cases Commentary

Due to the word count limitation of the article and in order not to lose its flow and purpose, the cases’ presentation was brought down to these three, although there were many more worth presenting. Therefore, the bottom line and common characteristics for them all should be stated. In brief, there is a consensual collective approach to patients suspected or confirmed for SARS-CoV-2 infection in this primary health care institution. According to it, the initial approach should be to individually and orientationally access the most likely course and prognosis of disease severity for every single patient at the very start. In order to obtain a proper assessment, every patient is first looked through a prism of main characteristics that are known as important COVID-19 prognosis factors such as age, sex, comorbidities, and BMI. Additionally, findings collected by anamnesis, physical examination (lung auscultation including), and diagnostic tests such as laboratory analysis and X-ray imaging are summed up to get the full picture and final assessment of every patient. Based on the assessment, the patients are roughly divided into low, intermediate, and high-risk groups, which then determine further therapeutic management. In particular, patients considered as with low risk of developing a serious disease (which are usually young, healthy people, and especially female) usually receive just symptomatic therapy and are advised to inform the doctor if their condition is progressively worse, if they experience “jump” rise in body temperature, any breathing difficulties, or unusual symptoms, or if the symptoms last for more than 5–7 days. From our experience, patients assessed as low risk usually do not need follow-up encounters except if they notice something unusual in their general condition. The next group comprises patients accessed as intermediate risk, most often middle-aged men with or without comorbidities. They usually receive just symptomatic and supportive therapy but are strongly advised to have a control encounter in a couple of days to check up and adjust the therapy if needed. In many cases, they do develop pneumonia which is usually not that extensive and is pretty much well controlled with azithromycin. If pneumonia developed, they are urged to have another control appointment(s) and usually additionally receive aspirin (firstly in higher doses and then prophylactic antiaggregating dose for a month). Many of them are prescribed with additional n-acetylcysteine 600 mg daily as long as pneumonia lasts. Some need an additional antibiotic if later signs of bacterial superinfection are noticed. Finally, the most “demanding” are high-risk patients, which usually are mature adulthood men and especially age group between roughly 55–75 years with or without comorbidities, as well as obese patients of practically any age. Once accessed as “high risk,” they usually receive azithromycin 500 mg daily for 5 days, n-acetylcysteine 600 mg daily, and aspirin (usually in higher doses in the first few days, except if they already have an anticoagulant in their therapy), alongside symptomatic and supportive therapy. These patients require almost everyday control check-ups, especially in the disease’s phase when they start to deteriorate, which is usually due to extensive bilateral pneumonia progression. At this point, the parenteral therapy is started with intravenous fluids and dexamethasone (along with gastroprotective therapy) if the earlier criteria for its administration are met. If there are signs of significant bacterial coinfection, intravenous antibiotics are also received. Indeed, as suggested by the relevant literature, it is common that patients who have antibiotics in their therapy are prescribed additional probiotics due to their supposed favorable properties (Bottari et al., 2021).

Patients who meet the WHO (not the NIH) criteria of severe COVID-19 (World Health Organization, 2020a) are suggested for hospitalization. However, very often patients refuse hospitalization despite the serious deterioration of their health. In these cases, the doctor is obliged to manage the patient as if he is a clinician. This usually requires supplemental oxygen for a patient at least for a few hours daily, and even other additional measures such as low-molecular-weight heparin administration where indicated. These patients are encouraged to do breathing exercises daily and are advised to spend as much of their time in sitting and standing positions so the lungs can ventilate properly. For them, special rooms are arranged as part of the health center in a form of a “daily hospital” where they can stay for an hour to two receiving their therapy. It is crucial that the same doctor follows, monitors, and treats the patient throughout the entire disease process. Of course, the doctor is always consulting his colleagues regarding patient condition if needed. From our experience, almost all of these patients treated exclusively in our primary health center had beneficial outcomes, speaking of their overall wellbeing, taking into count expectations of general condition and health status after overcoming COVID-19. These aspects will be further discussed.

## Discussion

Despite the fact that more than a year has passed since the COVID-19 is present in our worldwide society, and although vaccination against its causative agent already started in many countries, we witness that this disease still represents a major global concern even in the first part of 2021. By looking into daily reports of newly confirmed cases and deaths, as well as trends with vaccination, the chances are it will continue to stay so for a while longer. This is especially true for developing countries where the pandemic’s toll is even higher, which is also seen here in Bosnia and Herzegovina and is mostly due to poor economic status (meaning weaker health system as well) and less accessible vaccines. Another key factor that must not be overlooked is the continuous emergence of mutant strains of SARS-CoV-2, which threatens the efficacy of the developed vaccines. Additionally, having in mind the high prevalence of the disease and also relatively high mortality associated with it, despite the advances in its treatment and management, it is still reasonable and even crucial to discuss the ways of improving outcomes of the patients suffering from it (World Health Organization, 2020a; Chowdhury et al., 2020; Gautret et al., 2020; Sorci et al., 2020; Beyrer, 2021; Kovacevic et al., 2021; Lambert, 2021).

**FIGURE 4:**
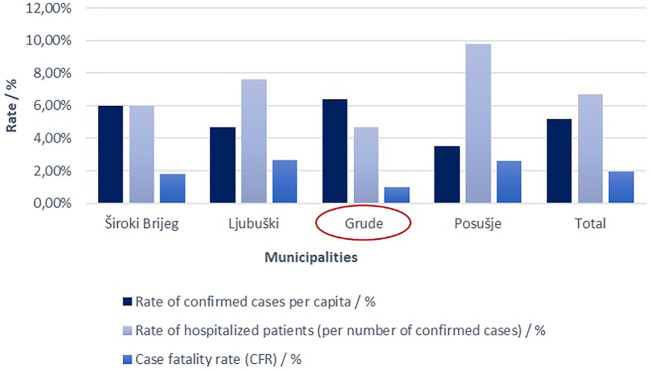
Graphical representation of data showed in Supplementary Table 1. Accordingly, the municipality of Grude has the far lowest rate of hospitalizations (4.67%) as well as mortality rates (CFR) from COVID-19 (0, 99%), despite having the highest rate of confirmed cases *per capita* (∼6.4%).

Speaking of outcomes of patients treated from COVID-19, there is a strong need that all the data, experiences, and findings of medical professionals in charge of these patients are shared with the worldwide healthcare community in order to improve the management and consequently the outcomes of these patients. Hence, the results achieved here at Grude Health Center, with the CFR below 1% for the Grude municipality comparing to the county’s CFR of nearly 2% and the one on a country’s level of around 3.7% (all as of December 31, 2020), definitely should be shown and discussed. To mention, the municipality of Grude belongs to West Herzegovina County where there are in total four primary health care centers (one on each municipality), without any other higher-level health institution. Thus, all patients requiring hospital admission are sent to the neighboring county’s university clinical hospital. Anyway, regarding local COVID-19 statistical data, the county’s official data (also as of December 31, 2020) were kindly provided by the Institute for Public Health of West Herzegovina County (shown in Supplementary Table 1). As clearly seen from Supplementary Table 1 and belonging graphical representation (Figure 4), the municipality of Grude has the far lowest rate of hospitalizations as well as mortality rates (CFR) from COVID-19, despite having the highest rate of confirmed cases *per capita*. To illustrate, there was only one out of every 21 COVID-19 patients hospitalized from Grude compared to 1/16 from Široki Brijeg, 1/13 from Ljubuški, and 1/10 from Posušje. Further, when comparing COVID-19 mortality rates among the municipalities, the CFR is almost half the rate for Grude than for Široki Brijeg and even more than two and a half times lower compared to Ljubuški and Posušje. Another meaningful comparison may be drawn toward neighboring counties (Herzegovina-Neretva County and Canton 10) since all patients from these three counties gravitate toward the same tertiary health care facility. Thus, again by the end of 2020 (as of December 31st), the CFR in the Herzegovina-Neretva County was around 1.2%, while it was around 2% in the Canton 10, which is still noticeably higher than that in the municipality of Grude (Institute for Public Health of Federation of Bosnia and Herzegovina, 2020).

Considering all presented data and earlier described general approach to COVID-19 patients as well as particular in detail real cases examples, it could easily be assumed that the management of these patients, which is practiced at Grude Health Center, may be beneficial and may result in better outcomes. Briefly, this management implies watchful care of those COVID-19 patients who may deteriorate or are deteriorating, in the form of adequate peroral therapy, regular check-ups, and case-to-case customized medical interventions of a single physician for each patient. The physician consults if needed other colleges during the process of managing each patient. Medical interventions performed during the treatment process are expanded, if needed, from the ones on a primary health care level to those on a secondary health care level in the form of “daily hospital.” The emphasis here is on well-timed parenteral dexamethasone or other approved corticosteroid treatment initiations, intravenous fluids, oxygen therapy, and parenteral antibiotics (if needed on a case-to-case basis). These interventions are performed at the same primary health care institution and under the same physician’s supervision. In order to achieve all of this, particular work and spatial organization are required. This means that there should be specially allocated and equipped spaces for the care of these patients only, preferably with entrance to these spaces directly from outside (while bypassing the institution's shared interior spaces) or using dislocated spaces for this purpose only. In the end, each patient is managed in the primary health care institution until his supervising doctor, based on clinical judgment, decides that the patient met the criteria for hospitalization. This is usually if the patient first presented with advanced disease or is deteriorating rapidly (usually in a matter of days), or there are signs of serious complications such as pulmonary thromboembolism. Of course, an individualized and rational approach is respected in the case of every patient.

Based on the achieved results, the authors of this study believe that COVID-19 patients may benefit more from a “home stay-daily hospital” combined approach than from an early hospitalization. The authors also believe that each day more spent surrounded by family with the ability to move around home freely and to take some fresh air and sun and each day less in the “COVID hospital” can bring overall better outcomes for these patients. In other words, the more days the severe COVID-19 patient spends under the primary health care system supervision in the manner described here, avoiding the “COVID hospital” if possible or at least by postponing hospitalization, the better the outcomes of the treatment process could be achieved. So, the idea is that the hospitalization threshold should not be as high and that even severe COVID-19 patients should be given a chance to continue treatment in the primary care settings for as long as possible (Levine et al., 2020). These thoughts should be especially considered if the adverse effects of patient isolation, which is often practiced and seen in “COVID departments,” are taken into count (Abad et al., 2010). More recently, even the idea of “Virtual Hospital” shows up as a positive model in managing the COVID-19 patients, thus supporting our reflections as well (Sitammagari et al., 2021). Another point is that these patients are probably “overtreated” in many cases, especially in terms of antibiotics use, which requires critical considerations regarding possible harms of “overtreatment” and definitely should be further investigated. Unfortunately, there are not many studies regarding this issue, and to our best knowledge, no other studies discussed the benefits of a similar concept of therapeutic management of severe COVID-19 patients in primary care settings. Therefore, the additional rationale behind this concept as well as convenient and larger studies should be conducted in order to further determine its benefits.

Additional thinking is provided as follows. Since the treatment and management of these patients depend heavily on the severity of the illness, the authors of this study put to the table the idea of adding the term “subsevere” to the current WHO classification of disease severity on nonsevere, severe and critical (World Health Organization, 2020a). It would include those patients whose health condition started to deteriorate but have not met the criteria of “severe” illness yet. In that way, this simple modification could improve the approach to the treatment of these patients and consequently the outcomes as well.

To this end, even though this study provides solid evidence in favor of a more comprehensive approach and therapeutic management of severe COVID-19 patients in primary health care centers, especially in developing countries, it still may have some limitations. Namely, possible sociodemographic differences among compared territories as well as differences in testing protocols among medical centers of each compared territory may affect the CFR values. However, it can generally be considered that these factors are pretty much similar for compared territories and thus likely not affecting the CFR values to a significant extent. Another possible disadvantage of the study is that it does not provide information on the patient’s long-term outcomes, which could provide additional evidence of the suggested concept. Anyway, since this could be a whole paper for itself, the authors of this study plan to do so in the near future, leaving, for now, these positive results and experiences as preliminary evidence.

## Conclusion

To conclude, mild cases of COVID-19 are not the only ones that could (and should) be treated in primary health care settings, deteriorating and severe COVID-19 patients as well can be treated and managed successfully, as seen. It seems that the commitment, resourcefulness, courage, and professionalism of primary health care physicians are required to achieve beneficial outcomes for these patients. Well-timed and prompt pharmacological and other treatment options’ initiation and administration by the same physician throughout (and after) the disease’s course may “spare” deteriorating and even severe COVID-19 patient from the hospitalization and thus bring better overall outcomes. Our experiences and results suggest that these patients usually can be managed well in the primary health care all up until rapid (in a matter of days) or acute (in a form of a complication) deterioration of health condition occurs, or the patient first presents to a physician in an advanced phase of a severe disease, where the hospitalization is strongly recommended. The concept presented here could be applied especially for lower to middle resources countries where the health care system’s capacities are limited, requiring constant improvisations and adjustments to current conditions.

## Data Availability

The original contributions presented in the study are included in the article/Supplementary Material; further inquiries can be directed to the corresponding author.

## Abbreviations

AL, alanine transaminase; BMI, body mass index; MR, modified release; PCR, polymerase chain reaction; WHO, World Health Organization; XR, extended release; US, United States.
