# Supplementary material for: Beneficial Treatment Outcomes of Severe COVID-19 Patients Treated Entirely in Primary Care Settings With Dexamethasone Including Regimen—Case Series Report
Source: Front Pharmacol. 2021 Aug 12;12:684537. doi: 10.3389/fphar.2021.684537 (PMC8387596; doi:10.3389/fphar.2021.684537)
Supplement: Supplementary file 1 [file Table1.DOCX]

Supplementary Material

# Supplementary Figures and Tables

| Municipality | № of confirmed COVID-19 cases / n | Rate of confirmed cases per capita* / % | № of hospitalized patients / n | Rate of hospitalized patients (per number of confirmed cases) / % | № of deaths (exclusively from COVID-19) / n | Case fatality rate (CFR) / % |
| --- | --- | --- | --- | --- | --- | --- |
| Široki Brijeg | 1794 | ~6% | 108 | 6.02% | 32 | 1.78% |
| Ljubuški | 1327 | ~4.7% | 101 | 7.61% | 35 | 2.64% |
| Grude | 1114 | ~6.4% | 52 | 4.67% | 11 | 0.99% |
| Posušje | 725 | ~3.5% | 71 | 9.79% | 19 | 2.62% |
| Total | 4960 | ~5.2% | 332 | 6.69% | 97 | 1.95% |

**Supplementary Table 1.** The COVID-19 statistical data for West Herzegovina County (Federation of Bosnia and Herzegovina) as of December 31, 2020.
